# Human Cerebrospinal fluid promotes long-term neuronal viability and network function in human neocortical organotypic brain slice cultures

**DOI:** 10.1038/s41598-017-12527-9

**Published:** 2017-09-25

**Authors:** Niklas Schwarz, Ulrike B. S. Hedrich, Hannah Schwarz, Harshad P.A., Nele Dammeier, Eva Auffenberg, Francesco Bedogni, Jürgen B. Honegger, Holger Lerche, Thomas V. Wuttke, Henner Koch

**Affiliations:** 1 Department of Neurology and Epileptology, Hertie-Institute for Clinical Brain Research, University of Tübingen, Tübingen, Germany; 2 Department of Neurosurgery, University of Tübingen, Tübingen, Germany; 3 San Raffaele Rett Research Unit, Division of Neuroscience, San Raffaele Scientific Institute, 20132 Milan, Italy

## Abstract

Pathophysiological investigation of CNS-related diseases, such as epilepsy or neurodegenerative disorders, largely relies on histological studies on human post mortem tissue, tissue obtained by biopsy or resective surgery and on studies using disease models including animal models, heterologous expression systems or cell culture based approaches. However, in general it remains elusive to what extent results obtained in model systems can be directly translated to the human brain, calling for strategies allowing validation or even primary investigation in live human CNS tissue. In the work reported here, we prepared human organotypic slice cultures from access tissue of resective epilepsy surgery. Employing different culture conditions, we systematically compared artificial culturing media versus human cerbrospinal fluid (hCSF) obtained from patients with normal pressure hydrocephalus (NPH). Presented data demonstrates sustained cortical neuronal survival including not only maintenance of typical cellular electrophysiological properties and activity, such as robust action potential generation and synaptic connectivity, but also preservation of tonic and phasic network activity up to several weeks *in vitro*. As clearly delineated by immunocytochemistry, single cell patch clamp and extracellular recordings, we find that in contrast to artificial culturing media, hCSF significantly enhances neuron viability and maintenance of network activity.

## Introduction

Investigation of pathophysiological mechanisms of human neurodegenerative and other central nervous system (CNS)-related diseases as well as the development of new therapeutic avenues in first line relies on studies involving model systems that include cell culture systems and animal models. Often it is assumed that obtained data apply or can be extrapolated to human CNS but there are numerous examples given by pharmaceutical research and drug development demonstrating that there are significant differences, even posing the risk that newly developed drugs which proved to effectively target certain pathways in model systems will not show the expected effects in patients. One way of approaching this challenge is the use of embryonic stem (ES)^1^ or induced pluripotent stem (iPS)^2^ cell technology or cerebral organoids^3^, allowing engineering and growing of “humanoid” neurons *in vitro* for investigation and analysis. While such approaches are considered a huge step forward, at the same time they are controversially discussed regarding e.g. purity, subtype-identity and maturity of generated “humanoid” neurons. Furthermore, this technology currently does not provide the tools to rebuild entire complex human CNS tissue (including entire cortical layering and columnar organization), which would be crucial when working toward a deeper understanding of physiological characteristics of human CNS circuitry or of pathophysiological mechanisms contributing to the development of human CNS-related disease. Alternative approaches to understand diverse aspects of highly complex connectivity and structure of the CNS down to morphological and functional properties of single cells/neurons, altogether governing the functions of the human brain, include MRI imaging technology^4^ and elegant stimulation studies^5^ or electrophysiological measurements in acute tissue slice preparations^6–8^. Interestingly, some of these studies were able to identify striking differences between human and rodent neurons, confirming that a direct translation from the animal model to humans might sometimes be misleading^6,9^. While – as mentioned above – electrophysiological characterizations of “static” intrinsic properties of human neurons can be successfully achieved in acute slices or potentially iPS-derived “humanoid” neurons^10,11^, investigation of intermediate/long-term changes of neuronal properties/function, as for example mediated by certain neuroplasticity mechanisms or by specific gene mutations of interest, remain challenging. In studies based on rodent tissue the development of organotypic slice cultures has been successfully used to bridge the gap of *in vitro* to *in vivo* translation^12^, but at the same time the challenges regarding immediate transferability of rodent data to the human organism have remained unsolved. The establishment of optimized human organotypic cortical slice cultures as an *ex vivo* system seems very tempting in this regard. Such systems could address some of the mentioned questions by enabling the investigation of dynamic properties of human neurons (such as neuroplasticity) and potentially of other cortical cells including glia over a time period of days to weeks. Furthermore, such cultures could serve as a platform to directly explore the functional impact and pathophysiological mechanisms of defined mutations (for example of ion channels) and their role in the development of CNS-related disease, which could be studied in the context of human cortical networks by help of viral-mediated overexpression. In addition, organotypic cortical slice cultures can be used for drug discovery and preclinical studies since it provides a peerless opportunity to investigate the effect of substances to human neuronal networks that developed physiologically. It also offers a possible tool for testing neuroprotective agents to networks maintaining their cellular complexity as well as their anatomical integrity^13^.

While previous studies in organotypic cultures prepared from human post mortem cortical tissue demonstrated continued viability of many cells including neurons up to several weeks *in vitro* ^14–16^, additional neurobiological data indicated that under certain conditions cultures prepared from resected human cortical tissue may be subject to a severe injury response, involving proliferation of reactive cells as well as progressive neurodegeneration^16^. However, recent work by another group demonstrated that such processes potentially could be ameliorated using optimized complex defined artificial culturing media, enabling at least partial preservation of characteristic neuronal morphology and importantly also pathological electrophysiological activities of mainly but not exclusively subcortical limbic structures (hippocampus, subiculum) in organotypic cultures of adult human tissue^17^. Building on these data, we here demonstrate that long-term neocortical neuronal viability and robust electrophysiological single cell and network function can be preserved in human organotypic cortical slice cultures by using human cerebrospinal fluid as culturing medium. These cultures could serve as a platform enabling direct validation of data obtained in model systems including but not limited to ES-/iPS technology, rodent primary neuronal cultures, organotypic slice cultures, and *in vivo* approaches or even non-mammalian heterologous expression systems.

## Results

### Neuronal activity of human organotypic brain slice cultures cultured in human CSF vs. traditional media

**Figure 1:**
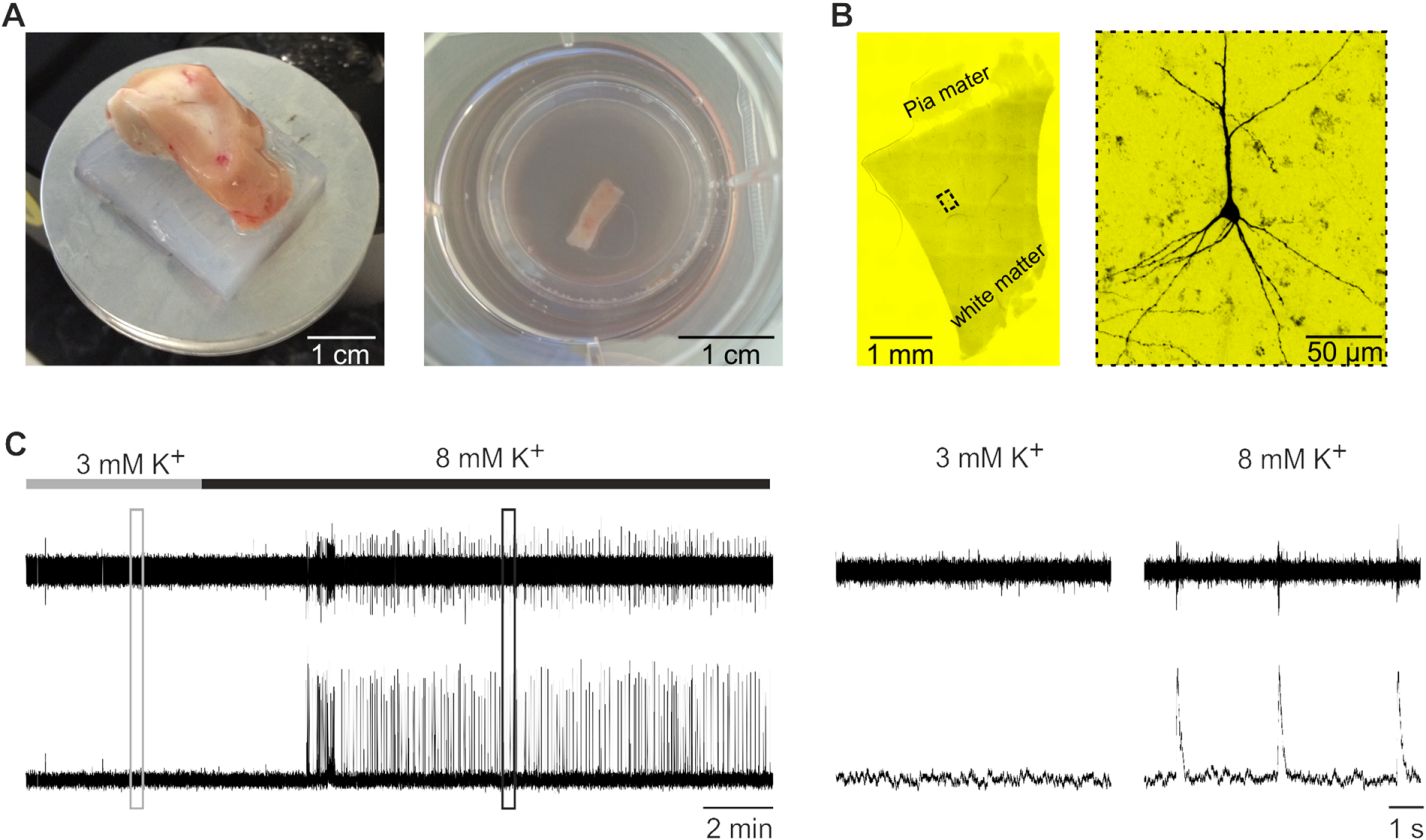
Human organotypic slices were cultured in six well plates for up to 21 days. (**A**) Slices were cut from tissue blocks perpendicular to the cortical surface (250–300 µm thick) and cultured in six well plates either in traditional culture media or human cerebrospinal fluid (hCSF). (**B**) Typical example of a human organotypic slice and a reconstruction of a neuron filled with biocytin after whole cell patch clamp recording revealing a pyramidal morphology (**C**) Multi-Unit extracellular population recording of a human organotypic slice culture, note the induction of rhythmic network discharges after increasing the extracellular K^+^ concentration to 8 mM (4 DIV). Boxes are magnified at the right.

**Figure 2:**
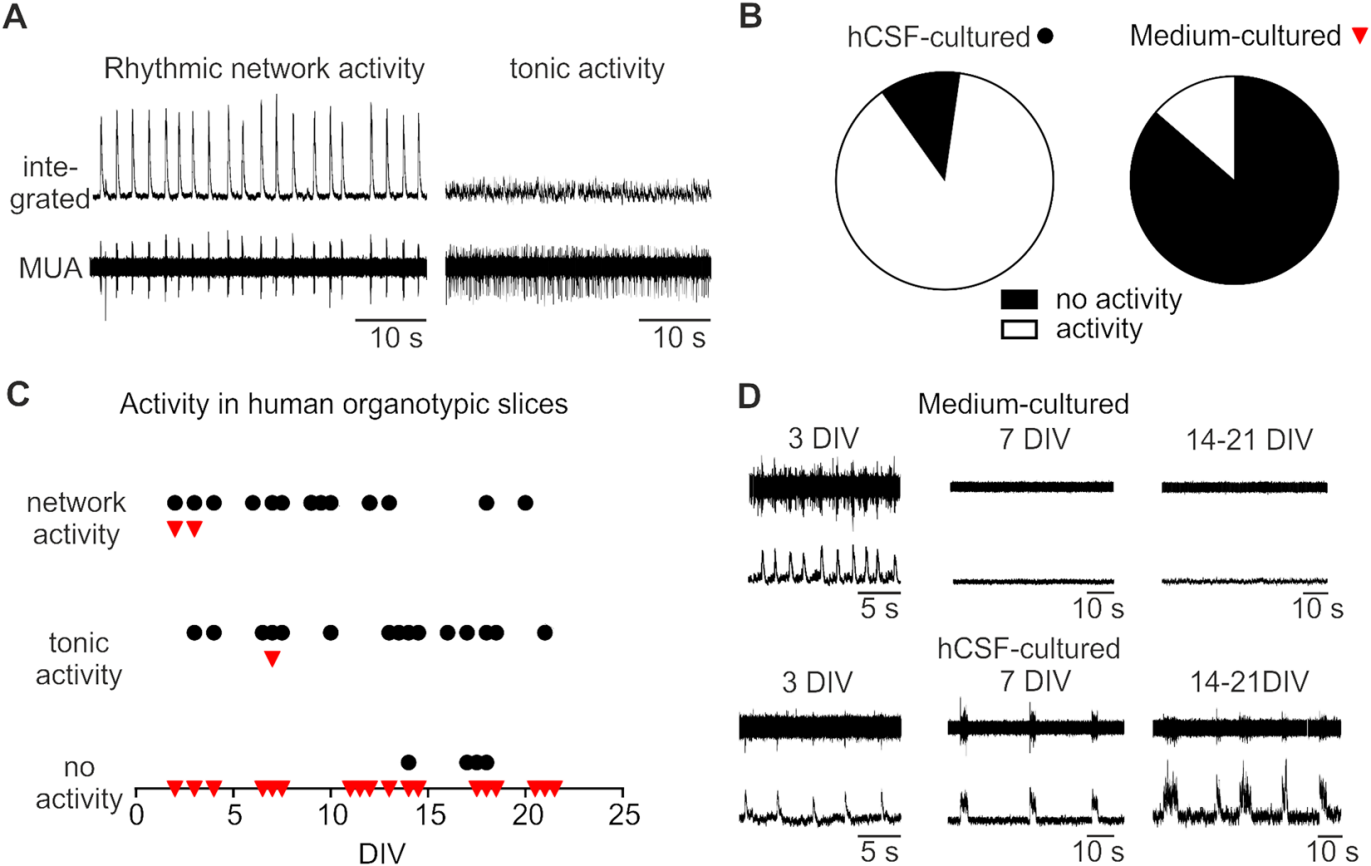
Electrophysiological recordings in human organotypic slice cultures. (**A**) In human organotypic slice cultures three main activity patterns were observed: Rhythmic network activity (A, left panel, 13 DIV), tonic activity (A right panel, 14 DIV) or no activity at all (D upper panels, 7 DIV and 14–21 DIV). (**B**) Overall, the number of slices being able to produce neuronal activity was much higher for slices cultured in hCSF (left pie chart) compared to slices cultured in traditional media (right pie chart). (**C**) Slices were recorded after different days *in vitro* (3–21 DIV). Slices cultured in hCSF (black circles) very frequently exhibited activity in comparison to slices cultured in traditional media (red triangles) (**C** and **D**). During the first days *in vitro* slices cultured in traditional media were still able to produce rhythmic and tonic activity, while in slices cultured for more than 7–10 days no rhythmic activity and just very rarely tonic activity could be recorded. In contrast, the majority of slices cultured in hCSF for 3–21 days showed either rhythmic or tonic activity (**B**,**C** and **D**).

From the initial tissue blocks, 250–300 µm thick slices (n = 57) were cut and kept in culture for 2–21 days either in human cerebrospinal fluid (hCSF) or in traditional culture media (either BrainPhys or organotypic slice culture medium (OSCM), see methods for details) before recordings were performed. To assess the viability of the slices, the electrical activity of the slices was tested with an extracellular population electrode to detect Multi-Unit-Activity (MUA). In all tested slices the concentration of potassium in the recording artificial cerebrospinal fluid (aCSF) was increased to 8 mM to facilitate spontaneous neuronal activity (Fig. 1C). In general, slices were found to either generate spontaneous rhythmic network discharges (Fig. 2A, left panel), tonic desynchronized firing (Fig. 2A, right panel) or no detectable activity at all (Fig. 2D, upper panels, 7 days *in vitro* (DIV) and 14–21 DIV).

The slices cultured in traditional media (see methods for details) showed only in a minority of cases (3/21) neuronal extracellular activity after being *in vitro* up to seven day*s*. Strikingly, only during the first three days of culturing, the slices kept in traditional media were able to produce network driven bursting activity (n = 2/21, 3 DIV; Figs 2B and D), while 1/21 produced tonic firing after 7 days *in vitro* (DIV). In all other slices tested no activity was detected (n = 18/21, 3–21 DIV; Fig. 2C). In contrast, the majority of slices cultured in hCSF produced either tonic activity or rhythmic network discharges (n = 32/36, 3–21 DIV; Fig. 2B–D). The 4/36 slices not exhibiting any neuronal activity were at least 14 DIV or even longer.

### Rhythmic network bursting in human organotypic slice cultures cultured in hCSF

**Figure 3:**
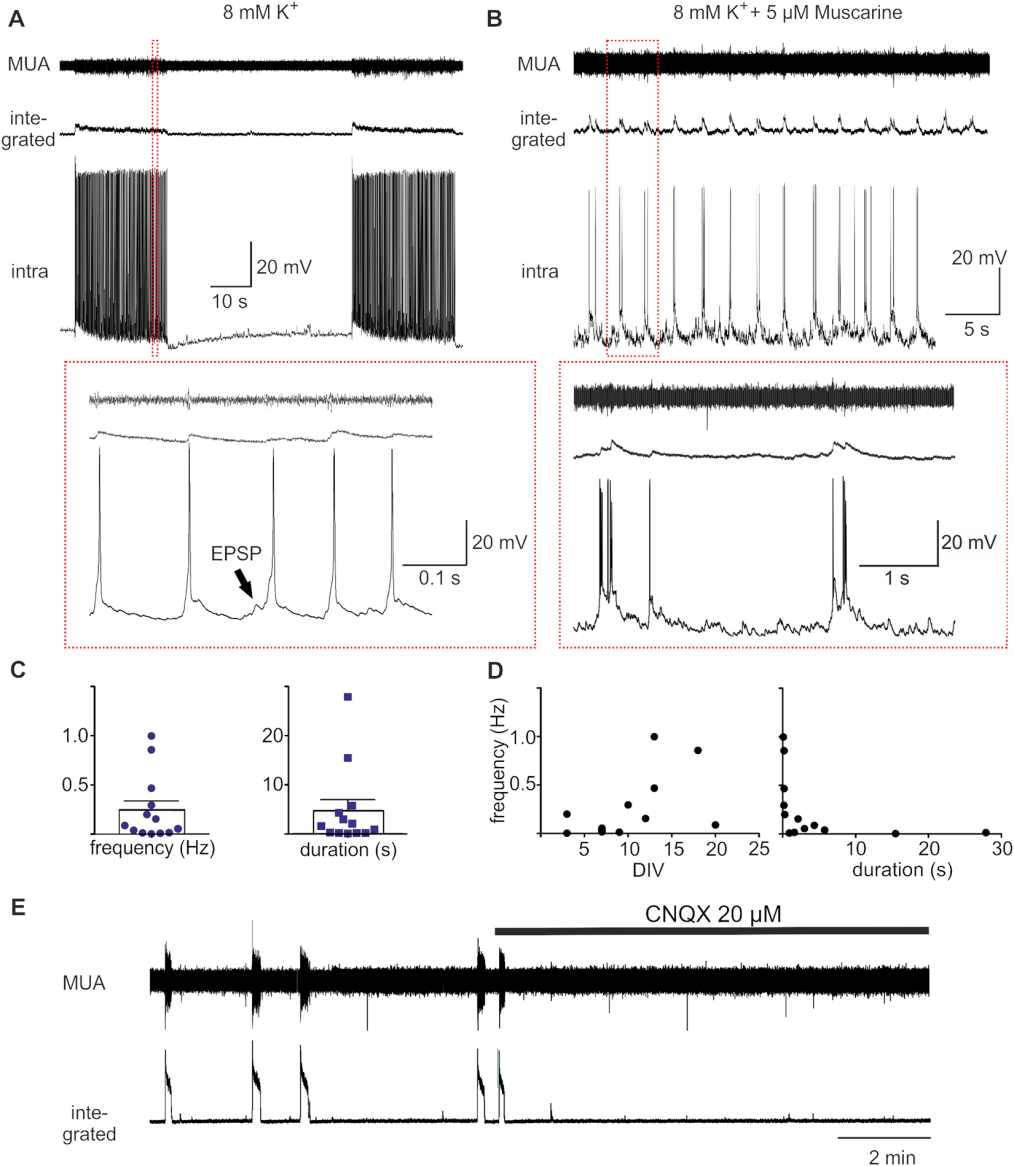
Rhythmic network activity of human slice cultures. The slices that produced rhythmic activity showed either long lasting network discharges (**A** and **E**) or oscillations with shorter bursts occurring with higher frequencies in the presence of 8 mM K^+^ (**D**). Shown are examples of the Multi-Unit Activity (MUA) recorded with an extracellular electrode and a simultaneous intracellular recording of a cortical neuron in high potassium alone (**A**) and in high potassium and the presence of Muscarine 5 µM (**B**). The red insets in A and B show expansions of the traces above. (**A**) Note that the action potential firing is riding on a barrage of excitatory postsynaptic potential (EPSP). The neuron in (**B**) was slightly hyperpolarized with a holding current of – 50 pA to emphasize the synaptic inputs. (**C**) Plot of the frequency of the oscillation and duration of all recorded network events in high potassium. (**D**) Plot of the frequency of network discharges against the DIV (left) and duration (right). (**E**) In a subset of the experiments (n = 3) the application of CNQX abolished the network discharges.

From the 32 slices that showed neuronal activity, 13/32 showed rhythmic network activity in aCSF with increased potassium concentrations of 8 mM. The network activity occurred with a mean frequency of 0.245 ± 0.09 Hz and had a mean duration of 4774 ± 2251 ms. The network activity seemed to be variable in duration and frequency (Fig. 3A–C). Some slices produced long lasting (up to 30 s) synchronous discharges (Fig. 3A), while others showed a higher bursting frequency with shorter durations (under 1 s). The frequency of these slices tended to be faster in correlation to the duration *in vitro*, but this was not statistically significant (Fig. 3D, p = 0.0739). In several slices (n = 11) we also tested the effect of bath application of Muscarine 5–10 µM on the network activity in addition to raising the extracellular potassium concentration to 8 mM. 5/11 slices showed tonic activity in the presence of 8 mM potassium, which increased in the presence of Muscarine, but only induced in 1/5 rhythmic network bursting. In 4/6 slices that showed rhythmic network bursting in 3 mM or elevated levels of 8 mM extracellular potassium, the application of Muscarine increased the frequency of bursting from 0.0029 ± 0.003 to 0.16 ± 0.29, while in two slices the application of Muscarine increased the tonic activity but stopped network bursting activity. To test if the network activity in human organotypic slices was driven by glutamatergic excitatory synaptic transmission we applied CNQX (20–30 µM, n = 3, Fig. 3E), which completely blocked the occurrence of network bursts in all tested slices.

### Firing properties of single neurons in hCSF and traditional culture media

**Figure 4:**
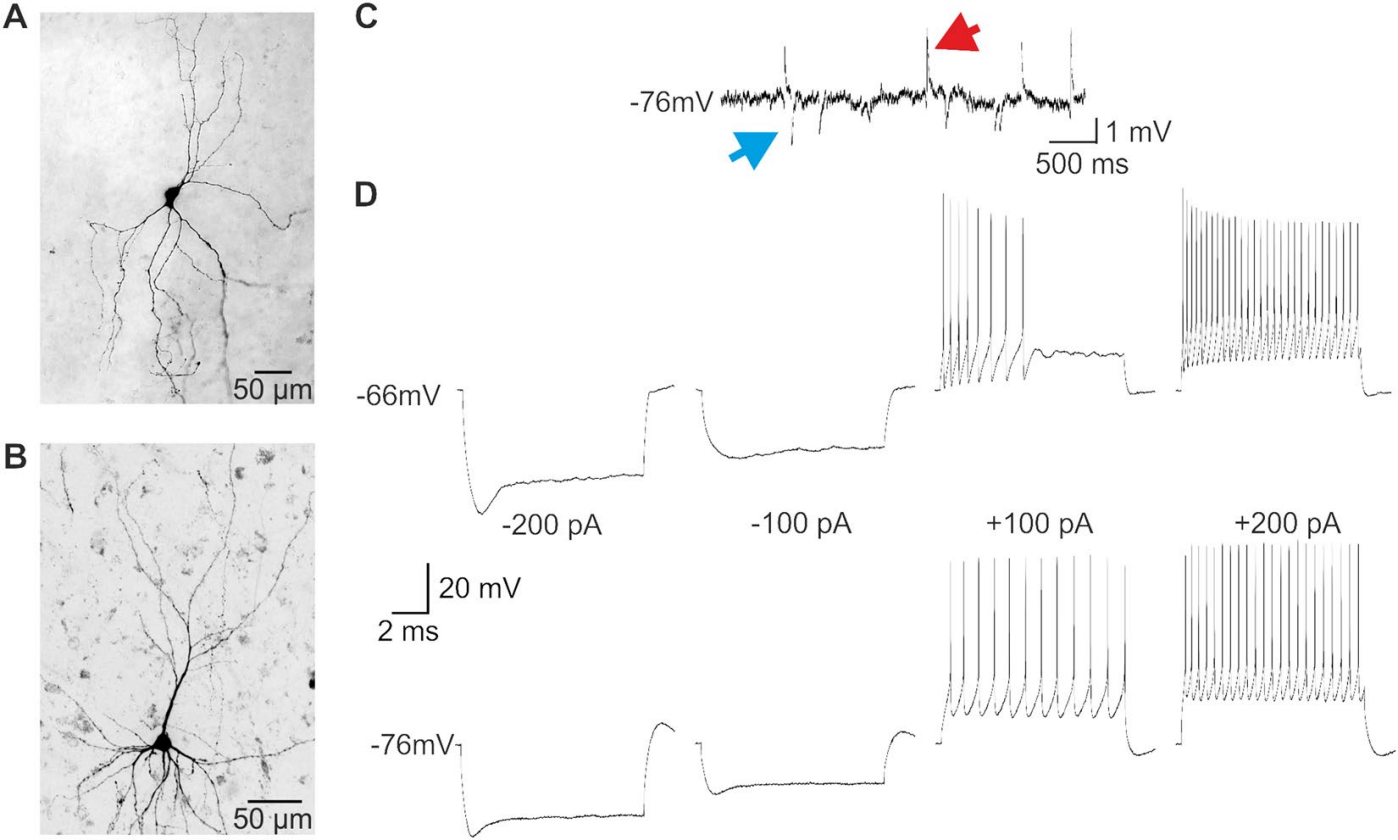
Representative examples of intracellular whole cell patch clamp recordings and biocytin filled morphology of human cortical neurons cultured in hCSF. In slices cultured in hCSF non-pyramidal (**A**, 9 DIV) and pyramidal neurons (**B**, 2 DIV) could be filled with biocytin and the response to hyperpolarizing and depolarizing current injections could be recorded. (**C**) The majority of cells recorded in current clamp received spontaneous inhibitory and excitatory synaptic inputs. Red arrow marks an excitatory postsynaptic potential (EPSP) and blue arrow an inhibitory IPSP in a cell recorded after 13 DIV. (**D**) All cells recorded in hCSF-cultured slices showed sustained action potential generation due to positive current injections (+50 pA to +200 pA) and showed typical firing behavior as reported before in human non – pyramidal (9 DIV, D upper trace) or pyramidal neurons (2 DIV, D lower trace).

**Figure 5:**
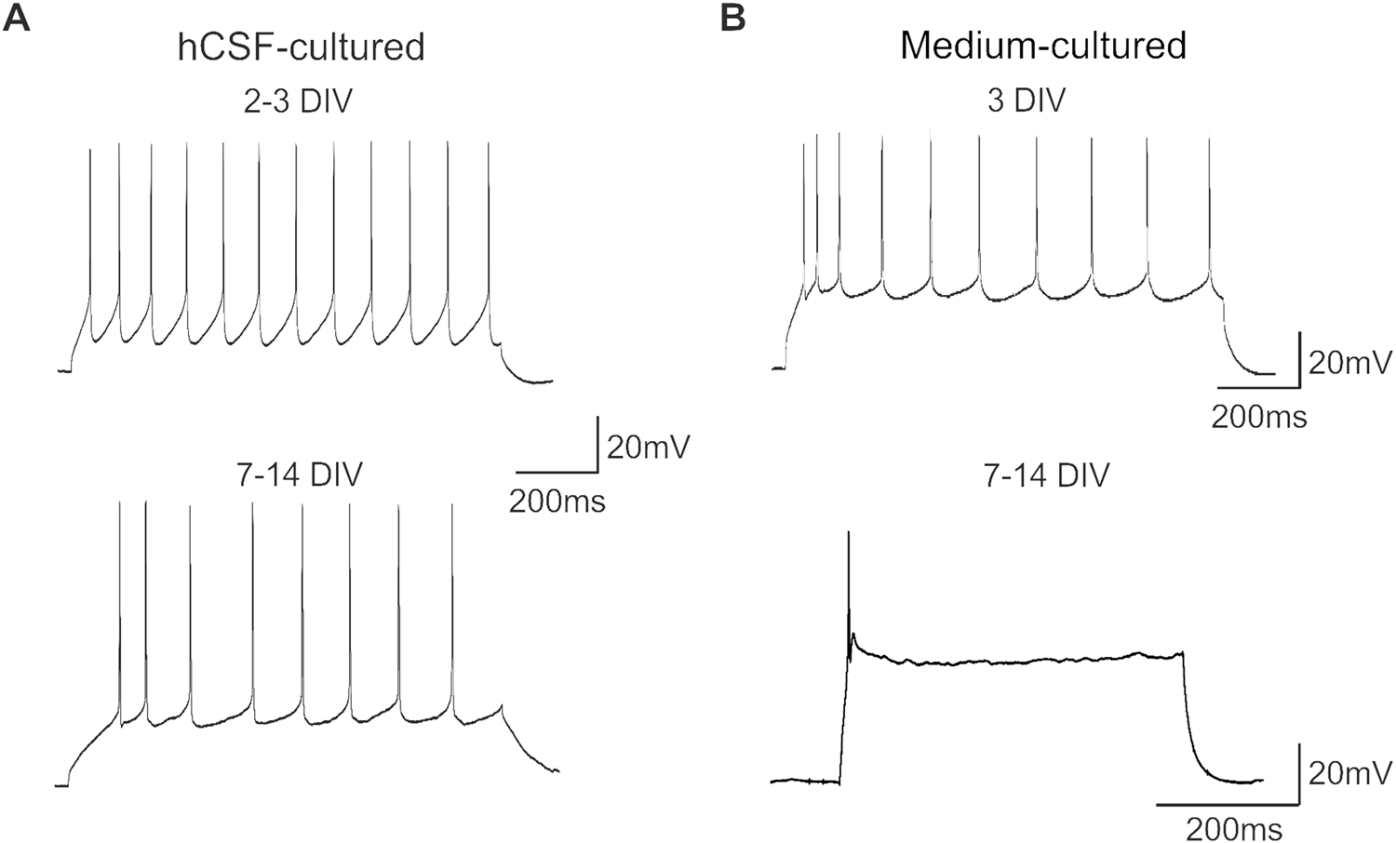
Effect of the culture medium on the excitability of human cortical neurons. The cortical cells recorded in slices cultured in hCSF (**A**) and after the first days in media (**B**, top) showed typical repetitive firing behavior to suprathreshold current injections (n = 10, 2–14 DIV for hCSF, n = 2, 3 DIV for media), while cells recorded from slices cultured in media for more than 7 days showed only a single action potential on suprathreshold current injections (**B**, bottom, n = 2).

In a next step, we investigated in a small number of cells, if neurons cultured in human organotypic slices were still able to produce firing behavior similar to the one of acute slices reported previously^7^. We recorded a total number of 14 cortical neurons in slices cultured in hCSF or traditional media at different time points (2–14 DIV, Table 1, Fig. 4). All neurons tested had a resting membrane potential more negative than −60 mV with an average of −70.3 ± 1.9 mV. Upon positive supra-threshold current injections all neurons cultured in hCSF (n = 10, 2–14 DIV) were able to fire repetitive trains of action potentials (Fig. 4D, Fig. 5A). In slices cultured in traditional media we were able to record four cortical neurons (3 DIV-13 DIV, Fig. 5B). Repetitive supra-threshold current injections (up to 400 pA) led only to the generation of a single action potential in cells recorded in slices cultured in media for 10 to 13 DIV (Fig. 5B, n = 2), while two neurons recorded after 3 DIV cultured in media were able to produce sustained action potential firing (Fig. 5B).

**Table 1:** Properties of cells cultured in hCSF. PY = pyramidal neuron, Int = Interneuron.

Number	Resting potential	Excitatory input	Inhibitory input	Morphology	DIV before recording
Cell 1	−72	No	No	PY	2
Cell 2	−72	No	No	PY	2
Cell 3	−76	Yes	Yes	PY	2
Cell 4	−63	Yes	Yes	No	3
Cell 5	−63	Yes	No	Int	9
Cell 6	−66	Yes	Yes	No	9
Cell 7	−80	Yes	Yes	No	13
Cell 8	−74	Yes	Yes	Int	13
Cell 9	−74	Yes	Yes	PY	13
Cell 10	−63	Yes	Yes	No	14

The anatomy of six biocytin filled neurons recorded in hCSF-cultured slices could be reconstructed after fixation. We used biocytin filling for a better morphological reconstruction on a single cell level comparatively to Map2 staining for dendritic integrity on population level. Four of these neurons had the morphology of pyramidal cells of Layer 2/3 and showed also a typical regular spiking pattern (Fig. 4B, D). Two of the neurons we reconstructed had the morphology of non-pyramidal neurons and showed a distinct fast firing pattern typical for non-pyramidal neurons (Fig. 4A and D). Four of the cells recorded in hCSF-cultured slices could not be reconstructed. Of the four neurons recorded in slices cultured in traditional media we could recover two which had the morphology of pyramidal neurons and showed a regular spiking pattern (Fig. 5B, 3 DIV). The other two neurons showed an atypical firing behavior with only a single action potential upon supra-threshold (Fig. 5B, 10–13 DIV). In all cells recorded in hCSF-cultured slices, we recorded up to 10 min of spontaneous activity and detected in 8/10 neurons recorded in slices cultured in hCSF excitatory postsynaptic potentials (Fig. 4C, red arrow) and in 7/10 cells inhibitory postsynaptic potentials (Fig. 4C, blue arrow). Moreover, in 3/10 cells recorded in hCSF-cultured slices (3 DIV, 9 DIV, 13 DIV) phasic network driven population input was detected in elevated concentration of potassium (8 mM, Fig. 3A and B). In the cells recorded from slices cultured in media 1/4 cell received phasic population driven input (3 DIV).

### Histology of slices cultured in hCSF vs. traditional media

**Figure 6:**
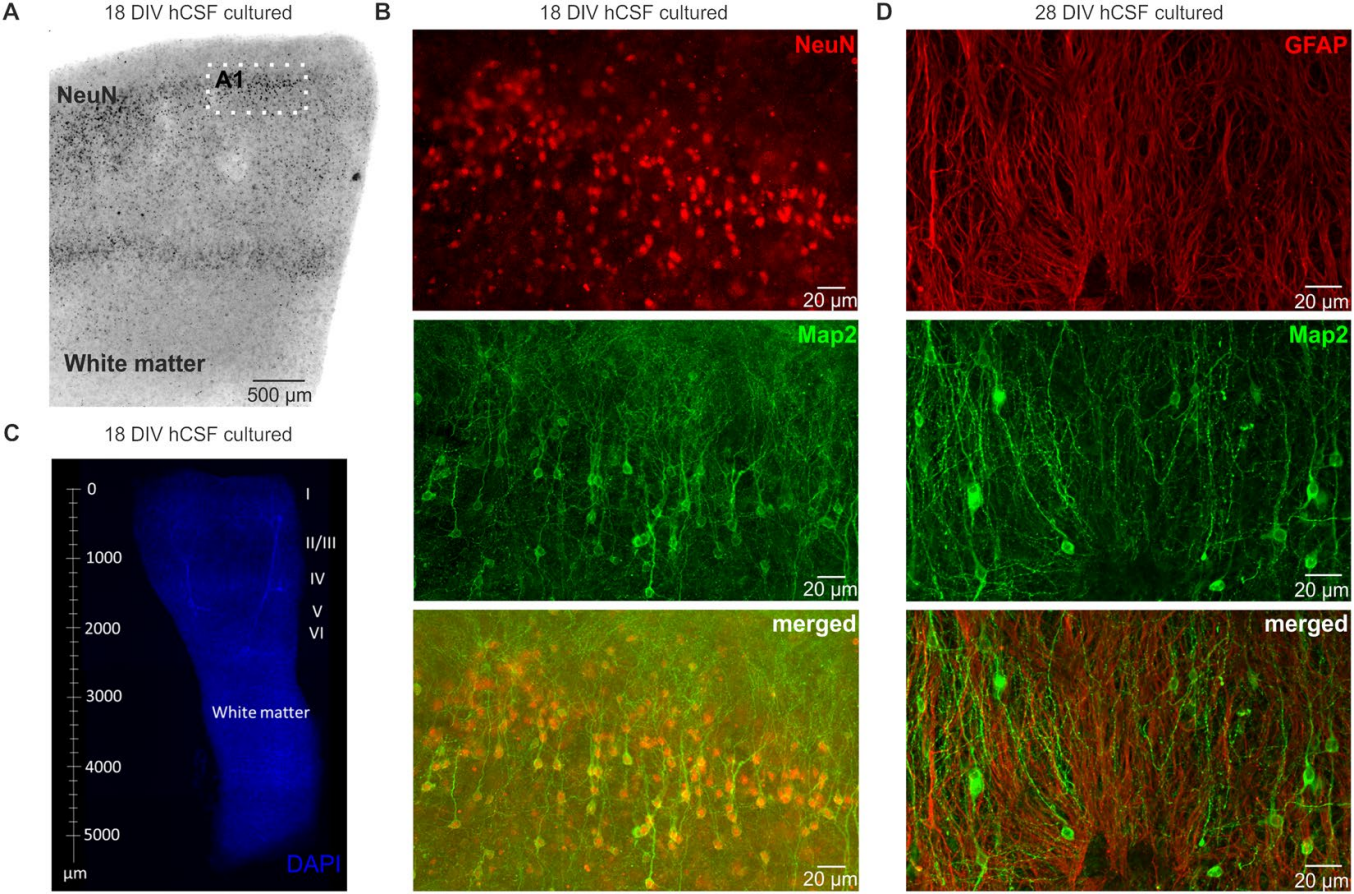
Morphology of human cortical slices in culture. (**A**) Example of a human organotypic slice after 18 DIV, note the dense labeling of NeuN positive cells in superficial layers and deep layers of the cortex. (A1) Inset of the superficial layers which are enlarged in (**B**) showing intact morphology of Layer 2/3 NeuN positive neurons with typical morphology of pyramidal cells revealed by Map2 staining and overlay. (**C**) Representative example of the size of a typical slice culture after 12 DIV (5 mm × 3 mm) with white matter and layers I–VI. **(D)** GFAP positive labeled astrocytes with Map2 positiv pyramidal cells cultured in hCSF after 28 DIV.

**Figure 7:**
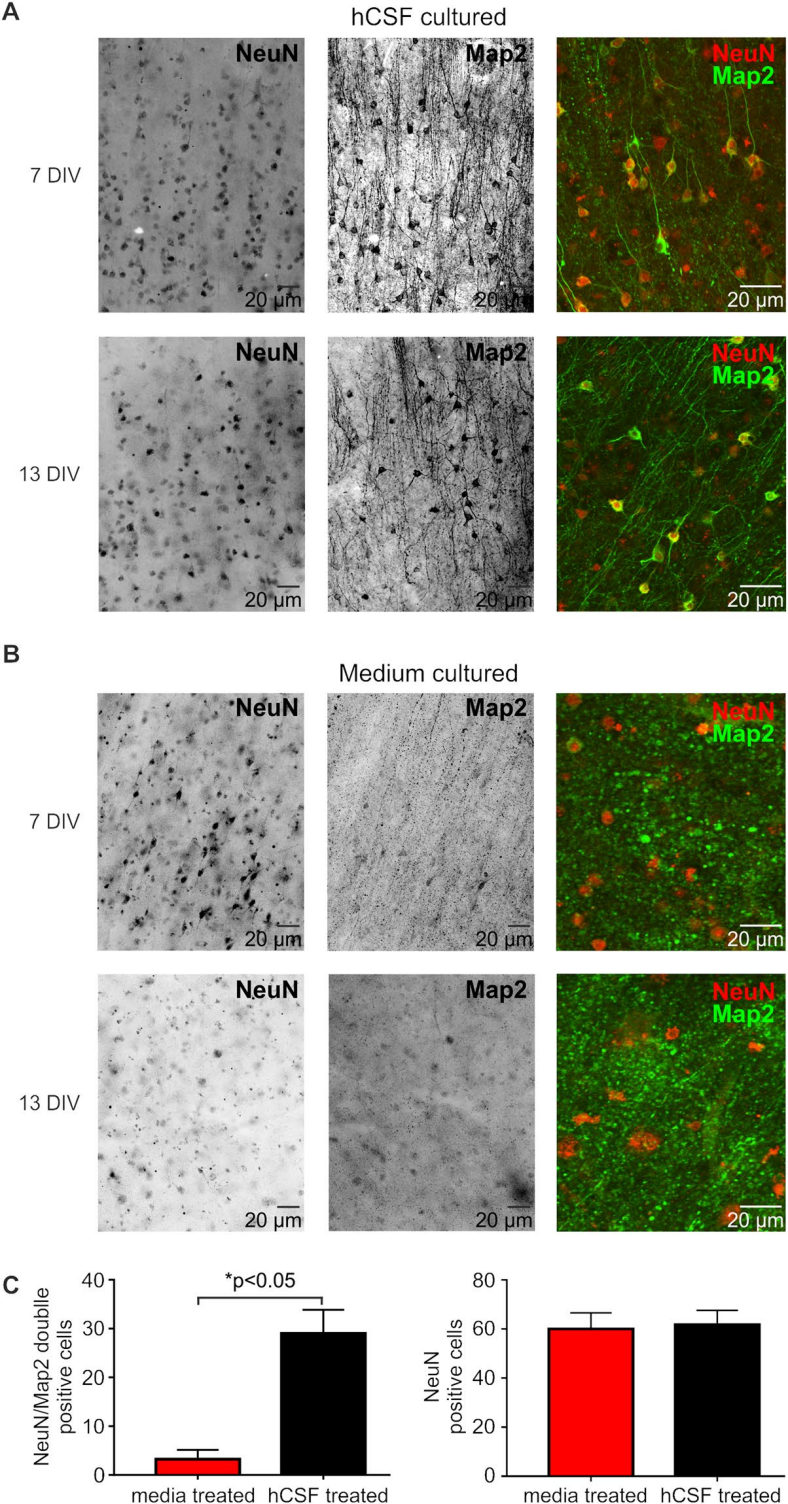
Morphology of human cortical neurons of slices cultured in hCSF vs. traditional media. Typical example of a NeuN and Map2 staining of human organotypic slices cultured in hCSF after 7 and 13 DIV (**A**) or traditional media after 7 and 13 DIV (**B**). Confocal images of the same slice showing co-labeling of NeuN and Map2 and intact morphology of the cells in slices cultured in hCSF (**A**) and loss of dendritic morphology in slices cultured in traditional media (**B**). Scale bars in all panels represent 20 µm. **(C)** Quantification of NeuN/Map2 double positive neurons in slices treated in traditional media compared to hCSF.

To determine if in addition to the electrophysiological changes also differences in the structure of the slices can be detected, we conducted a set of histological assessments. We used NeuN to stain the nuclei of post-mitotic neurons and Map2 to determine the dendritic morphology of the cells (Fig. 6A–D, n = 10 for hCSF and n = 4 for traditional media). After 3, 7, 10, 13 and 18 DIV in hCSF-cultured slices we found densely populated NeuN positive cells in all layers of the cortical slice (Figs 6B, 7A and B). Furthermore, the co-labeling with Map2 revealed an intact somato-dendritic morphology of the cells with typical ramification of the apical dendrite (Fig. 6B, 18 DIV, 7A and 7B, 7 and 13 DIV). In slices cultured in traditional media we found also a similar labeling of NeuN positive cells just after a few days (3 DIV and 7 DIV, Fig. 7C), but the Map2 staining revealed already a severe loss of the dendritic structures in the neurons of these slices (Fig. 7B). To quantify this data, we analyzed a representative area of n = 4 for traditional media and n = 4 for hCSF cultured slices at the time points 3 DIV, 7 DIV, 13 DIV and 18 DIV and counted the NeuN and Map2 double positive cells within a 450 × 450 µm field taken with a confocal microscope. We found a significant higher number of double positive cells in slices treated with hCSF compared to traditional media (Fig. 7C, Mann Whitney test, *p<0.05). However, the number of cells positive for NeuN alone in slices cultured with traditional media compared to hCSF was not significantly different. In addition, in slices cultured in hCSF, we tested if we could label astrocytes (n = 6; 3 DIV, 6 DIV, 7 DIV, 14 DIV and 28 DIV). We found at all stages dense labeling of GFAP positive cells in all layers of the human slices cultured in hCSF (Fig. 6D).

## Discussion

Organotypic slice cultures have been a useful tool in the last decades to study the physiology and pathology of neuronal networks^12,18,19^ or mechanisms of neurodegenerative diseases^20,21^. Here, we show that hCSF can enhance the survival and improve network function in human organotypic slices of adult humans for up to three weeks, including ongoing network and cellular function compared to traditional medium strategies. The advantage of slice cultures is that they stay within an artificial, but nevertheless physiologically developed, composition of neurons^22^, glia cells and even blood vessels^23^. In this, still in parts intact, original environment, the cellular and network properties of neurons can be studied in very controlled conditions. It should be noted, that in organotypic slices many artificial changes occur that lead to a different situation compared to acute slice preparations and to the situation *in vivo*. These changes include alterations in the synaptic communication between neurons^24^, a change of the intrinsic properties and morphology of the cells^12,24^ and an activation of the immune cells, due to the preparation of the slices and the culturing process itself. Keeping these caveats in mind, the slice cultures can be used to address and study processes that require a long-term observation and might be difficult to engage *in vivo*.

The human tissue that we have received for electrophysiological measurements was taken from patients undergoing a surgery for a resection of an epileptic focus. We and other groups used tissue that needed to be removed in order to get access to the pathological area, rather than pathological tissue itself. Access tissue is not considered to be part of the epileptic focus. Nevertheless, the cells cannot be termed “normal human neurons”, since they have been part of an epileptic brain for many years or may have been altered by the presence of antiepileptic drug treatment. To address this problem, Verhoog and colleagues did a careful comparison of the basic properties of human neurons of epilepsy patients and patients with a tumor^7^. No significant differences were found in the basic parameters of the cells indicating that these parameters might reflect most likely the normal properties of neurons. Another important point that has to be considered when working with human brain slices is the variability of the tissue samples (age of patients, genetic background, taken medication, part of resected tissue). To properly address such factors intra-experimental controls are needed from the experimental tissue obtained from the same patients. In our study the culture treatment of the human slices with either hCSF or traditional medium led to a significantly different outcome, when starting from the same patient tissue. As an alternative, we and also other groups have started to use iPS cells from patients to study the physiology and pathology of human neurons. Also this approach seems to have limitations that need to be considered. Adult neurons of patients have undergone development within their natural network environment within the brain up to the time point of surgical intervention and subsequent culturing. In contrast, “human neurons” that were generated from iPSCs undergo an artificial development in a rather rapid time *in vitro* and may even differentiate into neuron subtypes, resembling identities that do not even physiologically exist within the human brain^25,26^. Therefore, studying mature human neurons in organotypic slice cultures as an experimental approach seems, keeping the limitations in mind, a promising and powerful tool.

However, even in the same culture conditions we observed a variability in the outcome of neuronal activity of the tissue. Most slices cultured in hCSF showed a tonic firing behavior in high potassium concentrations (8 mM), while a third of the slices showed synchronized network discharges (Figs 2 and 3) and a small number did not produce any activity. It stays uncertain what determines the outcome of the slices. Surely, not all slices prepared from the same patient have had the same starting quality, due to the position in the block or other external factors. Also the thickness and size of the slices might have varied slightly and therefore plays a role in the outcome. These differences were not studied in detail, but we plan in further experiments to test the outcome of different sizes and thicknesses of human slice cultures to optimize this tool. In addition, in a recent study by Anderson and colleagues physiological firing behavior in neurons of human organotypic slices cultured in a medium based protocol was reported^14^. However, the authors account that more than half of the neurons they recorded showed normal resting potential of approximately – 60 mV, but were not able to produce action potentials in response to depolarizing current injections^14^. Interestingly, these results are in line with our observations reported in this study, that in medium based culture protocols the neurons seem to be able to survive for up to 14 days, but show progressively reduced or abnormal firing properties (Fig. 5B). At this stage we have not performed a quantitative comparison of the firing properties between cells and cell types in acute and organotypic slices, which would exceed the scope of this study.

Taken together the data of our study provides strong evidence that human Cerebrospinal Fluid plays an indispensable role for the survival and physiological function of neurons. The main finding of this study is that human organotypic slices maintained in hCSF showed increased survival of neurons (Fig. 7) and intact network (Figs 2 and 3) and cellular function (Figs 4 and 5) compared to slices kept in media based culture methods. So far we have not directly assessed the survival of specific neuronal subpopulations (inhibitory vs. excitatory), but the occurrence of both spontaneous inhibitory and excitatory inputs suggests a substantial survival of both major cell types. It should be noted, that in this study we did not aim to detect or define the components in the used hCSF that could explain this effect. In the literature, there are numerous suggestions that various neuroactive molecules contained in hCSF could have modulatory effects on the neuronal behavior^27,28^. However, relatively little is known about the exact mechanism by which hCSF is modulating the neuronal activity and survival^29,30^. One mechanism, that could play a key role in the survival of neurons, might be the activity of the cells itself. It has been shown that inactivity of neurons can lead to apoptosis and increased cell death^19,31^. Indeed, several studies report that the presence of hCSF leads to increased activity of neurons due to an increase in the intrinsic excitability of cells, suggesting that the activity might play a role^29,32^. However, this might just be one possible mechanism. The CSF of humans is composed of a vast amount of compounds, ranging from ions, proteins, lipoproteins and metabolic products to neuropeptides and hormones^30,32^. It has been suggested that CSF even changes its composition and can act as mediator for signal transmission^33,34^. More strikingly, in states of disease, as for example during a viral or bacterial meningitis, the composition of CSF is changing dramatically and seems thereby directly influencing the function of the brain^35^. Taken together we propose that CSF of healthy individuals (or of normal pressure hydrocephalus patients) might represent a more physiological environment for human adult neurons providing these cells not only with nutrients and removing products of the neuronal metabolism, but also improving the electrophysiological function of the neuronal networks^33^. Further studies have to investigate more detailed which neuroprotective molecules within hCSF are responsible for the increased survival of neurons in human organotypic brain slices and if the activity is a crucial component of this process. Indeed, the fact that mature human neurons seem to depend on different components compared to rodent cells might help to discover specific neuroprotective factors, which might not be detectable in animal models. Once identified, these molecules might open up new therapeutic routes or explain failure of the predictions of animal models.

In this study, we show that human organotypic slice cultures maintained in human cerebrospinal fluid show an electrophysiological and morphological prolonged survival in comparison to slices cultured in defined culture media. This is reflected by improved single cell and network activity of these slices compared to traditional media based approaches and by an increase of the survival of adult human neurons within their original environment and with parts of the physiological network intact. Additionally, neurons of hCSF-treated organotypic slice cultures show a significantly higher number of neurons maintaining expression of the somatodendritic marker Map2 over an extended period of time (up to 18 DIV) in comparison to neurons of slices cultured in conventional media, indicating enhanced structural and morphological neuronal integrity and survival. Although the components of hCSF that are responsible for this effect are not identified yet and demand further examination, our results suggest that there might be striking differences in the components needed to culture human vs. rodent-derived CNS tissue. We propose that the human organotypic slice cultures provide a unique tool to study physiological and pathological mechanisms in mature human neurons. While clearly more work is needed to further investigate the cellular and network properties and the composition of the cells in human organotypic slices, this study provides a substantial step forward to bridge the gap between animal models and human physiology. In future studies 3D-reconstructive-confocal-microscopy based morphometric analysis (taking into account parameters such as length, number, and branches of processes, size of cell bodies and nuclei) of other cell types (e.g. astrocytes)^36^ may prove as a valuable tool to study overall tissue and circuit integrity from a morphological angle and therefore also a possible difference in the activation of astrocytes (for review see ref.^37^), complementing and extending the presented network and single neuron electrophysiology data and analyses of neurons via immunocytochemistry and biocytin filling.

Human cortical slice cultures may serve as a platform enabling investigation of certain mechanisms and components of neuronal network plasticity, long-term effects of novel chemical compounds relevant to drug development, or studies involving viral mediated overexpression of proteins of interest.

## Methods

### Patients

Human neocortical organotypic slice cultures were prepared from access tissue obtained from patients undergoing resective epilepsy surgery. As described before, frequently removal of cortical tissue outside the epileptic focus is required in order to get access to the pathology^7^. For this study, we collected and included data of seven patients (Table 2). All patients were surgically treated for intractable epilepsy, in one patient the histology revealed a low grade tumor (Gangliogliom WHO Grad I).

**Table 2:** Patients included in this study.

Patient	Age at surgery surgery	Gender	Resected brain area
1	38	W	parietal lobe
2	10	W	temporal lobe
3	31	M	temporal lobe
4	33	W	temporal lobe
5	24	M	temporal lobe
6	42	W	temporal lobe
7	17	M	temporal lobe

### Preparation of slices from patients

Approval of the ethics committee of the University of Tübingen as well as written informed consent was obtained from all patients, allowing spare tissue from resective surgery to be included in our study (# 338/2016A). All experiments and methods were performed in accordance with the relevant guidelines and regulations. Tissue preparation was performed according to published protocols^7^. Cortex was carefully microdissected and resected with only minimal use of bipolar forceps to ensure tissue integrity, transferred into ice-cold artificial (a)CSF (in mM: 110 choline chloride, 26 NaHCO_3_, 10 D-glucose, 11.6 Na-ascorbate, 7 MgCl_2_, 3.1 Na-pyruvate, 2.5 KCl, 1.25 NaH_2_PO_4_, und 0.5 CaCl_2_) equilibrated with carbogene (95% O_2_, 5% CO_2_) and immediately transported to the laboratory. Tissue was kept submerged in cool and carbogenated aCSF at all times. After removal of the pia, tissue chunks were trimmed perpendicular to the cortical surface and 250–350 µm thick acute slices were prepared using a Microm HM 650 V vibratome (Thermo Fisher Scientific Inc).

### Human organotypic slice cultures

After the cortical tissue was sliced as described above slices were cut into several evenly sized pieces (~1.0 cm × 1.0 cm, Fig. 1A). Subsequently, the slices were transferred onto culture membranes (uncoated 30 mm Millicell-CM tissue culture inserts with 0.4 µm pores, Millipore) and kept in six–well culture dishes (BD Biosciences). The plates were stored in an incubator (ThermoScientific) at 37 °C, 5% CO_2_ and 100% humidity. For electrophysiological measurements slice cultures were transferred into the recording chamber of a patch clamp rig. After finishing patch clamp- or extracellular recordings, slices were fixed in 4% PFA and processed for immunocytochemistry, as detailed in the Immunocytochemistry section.

### Traditional culture media

Human organotypic slice cultures were cultured in organotypic slice culture medium (OSCM) without serum as described by Eugène and colleagues^17^ or BrainPhys medium according to Bardy and colleagues^27^. OSCM medium was produced by adding select components (see Eugene *et al*. for details) to commercially available DMEM/F12 and BME media. BrainPhys is a serum-free neuronal medium with adjusted concentrations of inorganic salts, neuroactive amino acids, and energetic substrates (see Bardy *et al*. 2015 for details). The media was originally developed to improve the electrical and synaptic activity of mature human neurons. Both media were found to exhibit comparable effects on human organotypic slices in our experiments and respective data were pooled and will be referred to as traditional media.

### CSF collection

Human cerebrospinal fluid (hCSF) was collected from patients with normal pressure hydrocephalus (NPH). We received and pooled hCSF of several patients with NPH who needed to undergo a lumbar puncture as part of diagnostic or therapeutic procedures (up to 40 ml per patient). Approval of the ethics committee of the University of Tübingen as well as written informed consent was obtained from all patients. It is well established and known from daily clinical practice that hCSF of NPH patients exhibits physiological/normal hCSF chemistry values (lactate, glucose, cell count, protein levels – see Table 3) undistinguishable from the ones of healthy individuals^32^. The CSF was centrifuged at 4000 rpm at 4 °C for 10 minutes and the supernatant was collected and stored at −80 °C within one hour after lumbar puncture.

**Table 3:** Basic chemical parameters of pooled CSF from NPH patients.

		unit	normal range
Appearance	Clear		
Erythrocyte	<1	thousand/µl	
Nucleated cells	<1	1/µl	0–5
Leukocytes	<1	1/µl	0–5
Polymorphnuclear cells	0	%	
Mononuclear cells	0	%	
Lactic acid	2.2	mmol/l	0–2.2
Glucose	108	mg/dl	
Proteins	47	mg/dl	0–45

### Extracellular Multi-Unit recording

For extracellular and intracellular recordings slices were transferred into a recording chamber and continuously superfused with artificial CSF (aCSF) containing (in mM) 118 NaCl, 3 KCl, 1.5 CaCl_2_, 1 MgCl_2_, 25 NaHCO_3_, 30 D-glucose, and equilibrated with carbogen (95% NaH_2_PO_4_, and 30 O2–5% CO_2_, pH 7.4) in a recycling system. Temperature was maintained at 30 ± 1 °C. The signal contained multiunit action potential (AP) activity. Extracellular signals were amplified 10,000-fold and filtered between 0.25 and 1.5 kHz. To facilitate detection of network-bursts, this signal was rectified and integrated by an electronic integrator with a time constant of 50–100 ms. We used an extracellular amplifier Modell 1700 (Am-Systems) or NPI (Model Ext 10–2 F) and an integrator from NPI (Model INR-011).

### Whole-cell patch clamp recordings

Slices were positioned in a submerged-type recording chamber (Scientifica, United Kingdom/Warner apparatus), continuously superfused with aCSF and visualized with a BX61WI Microscope (Olympus) or a Leica stereomicroscope. Recordings were performed using recording electrodes with a resistance of 3–5 MΩ and filled with a whole-cell patch-clamp pipette solution containing the following components (in mM): 140 K-gluconic acid, 1 CaCl_2_*6H_2_O, 10 EGTA, 2 MgCl_2_*6H_2_O, 4 Na_2_ATP, and 10 HEPES, pH 7.2. The intracellular pipette solution contained biocytin (5 mg/ml) to allow for posthoc identification of the location and morphology of recorded neurons. Whole cell current-clamp recordings were obtained from cortical neurons using the visual-patch or blind patch technique. Whole-cell patch-clamp recordings were obtained with a sampling rate of 10 kHz and a low-pass filter setting of 2 kHz. Recordings were performed with unpolished patch electrodes manufactured from borosilicate glass pipettes with filament (Science products). Patch-clamp experiments were performed with a patch-clamp amplifier (Multiclamp 200B) or a NPI Bridge Amplifier (Model BA-01X), a digitizing interface (Digidata 1440 A or 1550 A Digidata), and pClamp 10 software (Molecular Devices). The junction potential was calculated and subtracted offline to correct the membrane potential in current clamp mode. After recording, the slices were placed in neutral buffered 4% PFA solution at 4 °C overnight for fixation followed by three rinses in PBS and by subsequent immunocytochemical staining procedures.

### Immunocytochemistry and image collection

For immunocytochemistry (ICC) slices were incubated for 60 minutes in goat block (PBS, 0.2% triton X-100, 5% normal goat serum) before primary antibodies were added in respective dilutions. Due to the thickness of the slices incubation in primary antibodies was performed overnight at 4 °C temperature and for 30 minutes at room temperature on the following morning. Slices were rinsed four times in PBS supplemented with 0.1% Triton X-100 and then incubated in Alexa-fluorophore-conjugated secondary antibodies in goat block (PBS, 0.2% triton X-100, 1% normal goat serum) for 1 hour and 15 minutes at room temperature. After four PBS rinses (2x in PBS supplemented with 0.1% Triton X-100 and 2x in PBS) the slices were stained with DAPI (1:5000 in PBS for 2–5 minutes). After four final rinses (3x PBS, 1x Ampuwa water) the slices were mounted in Fluoromount G (SouthernBiotech) on glass slides. Primary and secondary antibodies were used at the the following dilutions: mouse anti-NeuN IgG (1:100, Merck, ABN90), chicken anti-Map2 (1:500, Abcam, ab5392), rabbit anti-GFAP (1:250, Agilent, Z0334), goat anti-mouse IgG Alexa 568 (1:500, life technologies, A-11004), goat anti-rabbit IgG Alexa 568 (1:500, life technologies, A-11011) and goat anti-chicken Alexa 488 (1:500, life technologies, A-11039). Expression of tissue-specific markers was evaluated using a conventional fluorescenece microscope (Zeiss Imager.Z1) and laser confocal scanning microscopic analysis (Zeiss LSM 510 Laser Module, Zeiss Axiovert 200 M). Images were processed with Photoshop CS (Adobe Systems) and CorelDRAW (Corel Corporation).
